# Sensor-Oriented Path Planning for Multiregion Surveillance with a Single Lightweight UAV SAR

**DOI:** 10.3390/s18020548

**Published:** 2018-02-11

**Authors:** Jincheng Li, Jie Chen, Pengbo Wang, Chunsheng Li

**Affiliations:** 1 School of Electronic and Information Engineering, Beihang University, Beijing 100191, China; lijincheng_buaa@163.com (J.L.); chenjie@buaa.edu.cn (J.C.); lics@buaa.edu.cn (C.L.); 2 Collaborative Innovation Center of Geospatial Technology, Wuhan 430079, China

**Keywords:** path planning, lightweight unmanned aerial vehicle (UAV), synthetic aperture radar (SAR), aerial surveillance, A* search algorithm

## Abstract

In the surveillance of interested regions by unmanned aerial vehicle (UAV), system performance relies greatly on the motion control strategy of the UAV and the operation characteristics of the onboard sensors. This paper investigates the 2D path planning problem for the lightweight UAV synthetic aperture radar (SAR) system in an environment of multiple regions of interest (ROIs), the sizes of which are comparable to the radar swath width. Taking into account the special requirements of the SAR system on the motion of the platform, we model path planning for UAV SAR as a constrained multiobjective optimization problem (MOP). Based on the fact that the UAV route can be designed in the map image, an image-based path planner is proposed in this paper. First, the neighboring ROIs are merged by the morphological operation. Then, the parts of routes for data collection of the ROIs can be located according to the geometric features of the ROIs and the observation geometry of UAV SAR. Lastly, the route segments for ROIs surveillance are connected by a path planning algorithm named the sampling-based sparse A* search (SSAS) algorithm. Simulation experiments in real scenarios demonstrate that the proposed sensor-oriented path planner can improve the reconnaissance performance of lightweight UAV SAR greatly compared with the conventional zigzag path planner.

## 1. Introduction

Day and night airborne observation is a mission of great significance in both military and civil applications. Due to its low cost, lightweight unmanned aerial vehicles (UAV) has been widely utilized as an aerial surveillance agent in the recent decades, since it could provide comprehensive information of the environment using the onboard sensors [1,2,3,4,5,6,7,8]. To gather more useful information, the path of the aircraft is required to be designed specifically. This is termed the UAV path planning problem, i.e., designing a route for the aircraft from the starting position to the destination. Conventionally, the main considerations in UAV path planning are the feasibility, length, and safety of the flight path [9]. Nowadays, the cooperative missions such as target search [10,11,12], tracking [13] and surveillance [14,15,16] have been paid more attention by the UAV path planners.

The motivating problem of this paper is to monitor multiple dispersedly distributed regions of interest (ROIs) using a single lightweight UAV synthetic aperture radar (SAR). Due to its capability to provide day-and-night and weather-independent images, SAR has been widely used in earth monitoring [17,18]. The path of the radar platform is of significance for airborne SAR. Firstly, this is because motion of the airplane, the outcome of which is the flight path, is the essential solution in SAR [19]. Unlike the other equipments (e.g., daylight or infrared cameras) that gather information instantly at a fixed location, SAR monitors the environment via a virtual aperture formed by the continuous movement of the platform. In addition, the areas illuminated by the radar beam are mainly determined by the motion strategy of the radar platform. This is because the track of radar footprint is highly coupled with the route of the airplane, especially for the lightweight UAV SAR system without beam steering capability. Traditionally, the flight path of the airborne SAR can be designed as a straight line or a zigzag “mowing the lawn” pattern [20]. However, the lightweight UAV SAR is usually equipped with a narrower field of view on the ground due to its low flying altitude [15], making it unable to cover multiple dispersedly distributed ROIs in a single straight sweep. The zigzag path is also unsuitable due to the limited flight duration of the lightweight UAV. Therefore, a well-designed flight path is highly required for a lightweight UAV SAR system to enhance its spatial coverage performance.

**Figure 1:**
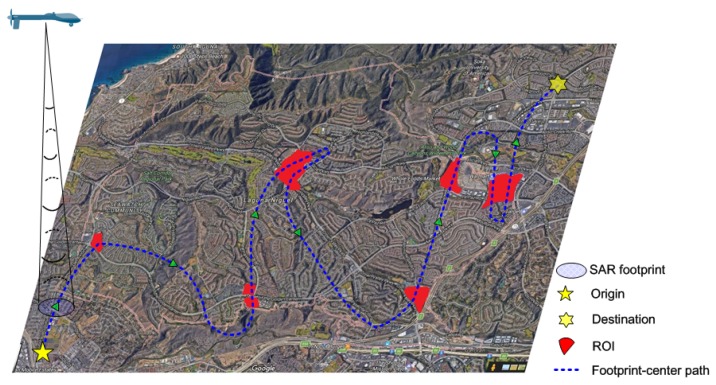
Illustration of multiregion surveillance by a single lightweight UAV SAR.

An instance of the sensor-oriented path planning problem addressed in this paper is illustrated in Figure 1. We aim to design a curved path for the UAV in order to cover all the interested regions. A number of factors should be taken into account by the path planner [9,21,22,23,24,25,26]. First, the physical limitations of the UAV and the full coverage of the ROIs should be satisfied. Meanwhile, the path costs in length and safety are desired to be as low as possible. In addition, we should consider the coupling between the UAV and the sensor’s footprint. Lastly, the unique motion requirements of the SAR sensor should be taken into account by the path planner.

With a variety of factors to be accounted for, the sensor-oriented path planning for UAV SAR under study is a constrained multiobjective optimization problem (MOP) in essence [21]. To handle the involved objectives and constraints, an image-based route planner is proposed in this paper. The main contributions of this paper can be listed as follows.

1. The SAR-oriented objectives and constraints on the flight path are analyzed in detail, and their mathematical expressions are presented in an image-based configuration space (C-space).
2. An image-based pretreatment method is developed to simplify the path planning procedure. By doing so, the path planning problem can be decoupled into two subproblems, i.e., locating the SAR-oriented path segments and designing the UAV-oriented sub-routes. Furthermore, the independent UAV-oriented sub-routes planning can be implemented in parallel to speed up the process.
3. A route planning method, named sampling-based sparse A* searching (SSAS) algorithm, is proposed to search near-optimal sub-routes effectively without breaking the constraints. In SSAS, we apply the sampling strategy to avoid the construction of search map, and we apply a bidirectional search scheme to provide the proper heuristic for the route planner.

The rest of the paper is organized as follows. We begin by presenting the related work in Section 2; Section 3 presents the problem statement; the proposed path planner is introduced in Section 4; Simulation results are shown in Section 5; and several conclusions and the discussion of future work are presented in Section 6.

## 2. Related Work

As a particular airborne SAR system, UAV SAR has been widely used in the civil and military applications such as environmental monitoring, terrain mapping, and aerial reconnaissance [5,20,27]. Compared with the traditional airborne SAR systems, UAV SAR is more flexible in conducting surveillance mission due to its safer operation, finer temporal coverage, and lower R&D expense [8,28].

Former researchers mainly focused on the system design [5,6,7,8,28], signal processing [29,30], and data application [20,27] in the study of UAV SAR. Spatial coverage is a key performance indicator for the airborne surveillance systems. Given the beamwidth of a UAV SAR system, the areas covered by the sensor footprint are mainly determined by the UAV path. However, seldom existing literature addressed the path planning problem in the research of UAV SAR. This is mainly because the early UAV SAR systems were generally mounted on the powerful drones with high flight altitude, heavy loading weight, and strong maneuverability [31]. Their swath can be large enough to cover several interested regions in a single sweep. In addition, the long flight duration and the beam steering technique could enhance their coverage ability further. However, the expenses of these SAR systems are quite huge, the alternative of which can be the lightweight UAV SAR [8,32,33]. Comparatively, the shortcomings of the lightweight UAV SAR systems are the narrow swath, short flight duration, and fixed beam orientation. To some extent, these disadvantages can be overcome by a well-designed route [21,28]. Sun et al. investigated the path planning for UAV SAR for the first time in [21]. The authors of [21] aim to design a route with fine imaging performance and acceptable path cost for a bistatic SAR system. However, only the spatial resolution of the interested targets is considered in [21], whereas the coverage performance is not accounted for.

Covering all points of an area is termed the coverage path planning problem [34]. However, we only aim to cover several dispersedly distributed ROIs, rather than the whole area, in this paper. Hence, the problem under study is more of an optimal path planning problem. Optimal path planning problem, which is not restricted to UAVs, has been intensively investigated in the domain of robotics [35,36,37,38]. The path planners aim to design optimal routes between two predefined positions for robots under various constraints. Searching the optimal UAV path is a non-deterministic polynomial-time hard (NP-hard) problem [39]. That is to say, the optimal route does not always exist, the search of which is computationally prohibitive and unnecessary even if it exists. Therefore, searching for a near-optimal path, with comparable cost but much less search time, is usually adopted in the existing UAV path planning algorithms [9,14,39,40,41,42,43,44,45,46,47,48,49,50,51,52].

The UAV path planning algorithms can be classified into the stochastic and deterministic categories. One of the most popular stochastic solutions is the evolutional algorithm (EA), which is proved to be flexible and efficient in solving the NP-hard problems [40,43,45,53]. As a type of bioinspired algorithm, EA simulates the evolution process in nature by selecting the candidates with better performance to produce the next generation. Other stochastic algorithms have also been applied successfully in UAV path planning, such as particle swarm optimization (PSO) [45,47,54], ant colony optimization [50,51], and gravitational search algorithm [52]. The main advantage of the stochastic methods is that they do not require constructing an explicit search map for the path planner, which is very time-consuming [43,44]. On the other hand, their deficiencies are the heavy computational burden, the unstable results in different runs, and the uncertain parameters that need to be tuned in different situations. In terms of the deterministic techniques, the designed route is stable if the parameters remain unchanged in different runs [14,39,41,49]. A real-time path planning algorithm named the sparse A* search (SAS) technique is proposed in [39]. SAS utilizes a sparse search tree to smooth the designed path. Moreover, a bucket-shaped area around the goal position is designed to achieve a predetermined approach angle to the destination. However, the map cost array used in [39] is inefficient to deal with the multiple objectives involved in the route planning. In addition, the update of the map cost array can be quite time-consuming [44].

Except for the feasibility, length, and safety of the flight path, the mission assigned to the UAV should also be considered in path planning. Tang et al. investigated the motion planning problem for a limited resource of sensor agents in an environment of multiple targets in [15]. In [15], the motion planning problem is interpreted as to find a time-optimal loop path to traverse the targets, which is solved by a gradient-approximation algorithm. However, the threats in the environment are ignored in [15]. Furthermore, only the point target is considered in [15], and the paths of the agent and the sensor footprint are not separated. Beard et al. addressed the multiple target coverage problem using multiple UAVs in [14]. Aiming at passing all the assigned targets, the path of each UAV is designed first in a threat-based Voronoi diagram. The Voronoi path is then smoothed through a fine trajectory generator to make the trajectory feasible within the dynamic constraints of the vehicle. Unfortunately, it is difficult to fit this method into the multiregion coverage problem addressed here. This is because the graph-based method can only be utilized when the targets can be seen as points in the map, i.e., the nodes of the Voronoi diagram. There is some other literature that is related to the problem studied here, such as [16,55,56].

## 3. Problem Statement

As aforementioned, the sensor-oriented planning problem studied in this paper is a constrained MOP in essence, which can be built as follows.

### 3.1. Problem Modeling

**Figure 2:**
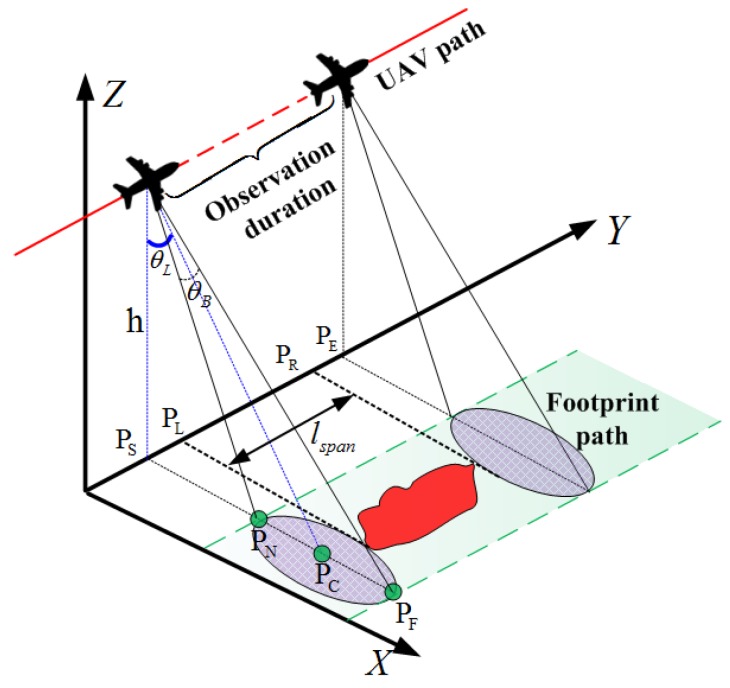
Observation geometry model of UAV SAR.

**Figure 3:**
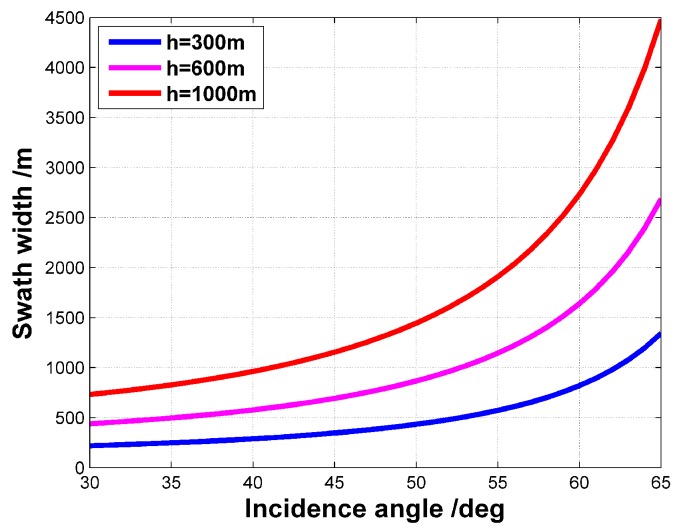
Swath widths of UAV SAR at different flight altitudes.

The observation geometry of UAV SAR is shown in Figure 2, from which the coupling between the UAV and the sensor footprint can be observed. For the lightweight UAV SAR, the stripmap mode is usually performed without either a squint angle or a steered beam [8]. Therefore, the beam pointing direction is fixed, and the beam center is vertical to the UAV path. The coverage capability of the SAR system is mainly manifested in the width of the ground swath, which can be calculated by

(1) $$\begin{array}{cc} l_{\mathrm{sw}} & =∥\vec{P_{N}P_{F}}∥\\ & =h\cdot \tan\left(\theta_{L}+\frac{\theta_{B}}{2}\right)-h\cdot \tan\left(\theta_{L}-\frac{\theta_{B}}{2}\right), \end{array}$$

where *h* is the flight altitude, $\theta_{L}$ denotes the incidence angle of the beam center, and $\theta_{B}$ denotes the elevation 3 dB beamwidth. Assuming the elevation 3 dB beamwidth is $30^{\circ }$, the swath widths at different flight altitudes are plotted with respect to the incidence angle in Figure 3. For the lightweight UAV SAR flying at 300 m, the swath widths are less than 500 m under most of the incidence angles. In these cases, the UAV SAR system is even unable to cover a single interested region (e.g., a stadium) in a single straight sweep, let along several ROIs that are sparsely distributed in the map. The possible solution could be the higher flight altitude or the larger incidence angle. However, the two alternatives mean the longer distances between the radar and the targets, which would require more powerful radar system with heavier weight. To enhance the coverage ability of the lightweight UAV SAR, a curved path with many more turns can be a sound solution.

**Figure 4:**
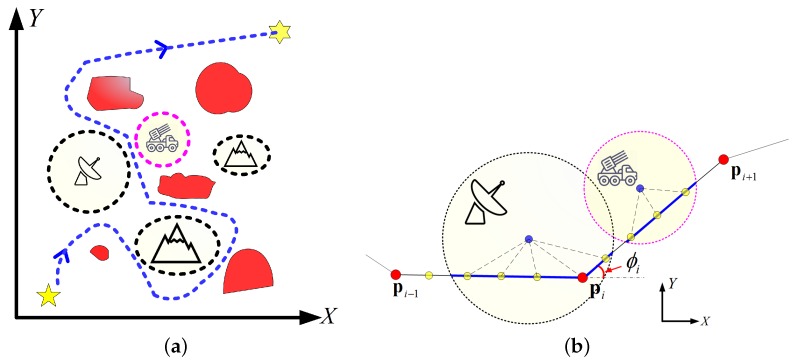
Illustration of the path planing in a 2D C-space. (**a**) the 2D C-space; (**b**) UAV path formed by connecting the waypoints.

The C-space for path planning is generally built in a 3D digital elevation map (DEM) or a 2D map image [47]. Considering that a stable path in the vertical direction is always desired for SAR image formation [19], a constant flight altitude is adopted by our path planner. In this way, the path planning for UAV SAR is simplified to be a 2D problem. A typical 2D C-space is illustrated in Figure 4a, where the *x*- and *y*-axes are pointed to the north and east directions, respectively. The locations of the significant elements in the environment can be marked in the C-space [9,44]. For instance, the ROIs are labelled in red. In addition, the threats and the terrain obstacles are marked by the dotted circles, which should be avoided by the UAV. The UAV route are generally represented by waypoints, which can be connected by a number of line segments [39,41,43,44] or a fitted curve [9,40,45]. As illustrated in Figure 4b, the former connecting way is adopted in our work for its simplicity and efficiency. Then, the task for the route planner is to optimize the position of the waypoints, which can be expressed as a constrained MOP, i.e.,

(2) $$\begin{array}{c} \min.\;J\left(P\right),\\ s.t.\sum_{j}c_{j}=0, \end{array}$$

with

$$P=\left[\begin{array}{cccc} p_{1} & p_{2} & \cdots & p_{N_{W}} \end{array}\right]=\left[\begin{array}{cccc} x_{1} & x_{2} & \cdots & x_{N_{W}}\\ y_{1} & y_{2} & \cdots & y_{N_{W}} \end{array}\right],$$

where $J\left(P\right)={\left[J_{1}\left(P\right)\;\;J_{2}\left(P\right)\;\cdots \;J_{N_{J}}\left(P\right)\right]}^{T}$ is the vector-valued objective, $c_{j}$ is a binary function ($c_{j}=0$ if the *j*th constraint is satisfied, otherwise $c_{j}=1$), $p_{i}$ denotes the two-dimensional coordinate of *i*th waypoint, $P\in R^{N_{W}\times 2}$ is the coordinate matrix, and $N_{W}$ is the number of waypoints.

The constraints and objectives oriented to UAV have been discussed thoroughly in the previous literature [9,43,48], so we put more emphasis on the unique motion requirements of the SAR system. In all, four constraints and three objectives, either SAR-oriented or UAV-oriented, are taken into account, the mathematical expressions of which will be presented in the following two subsections.

### 3.2. Constraint Functions

#### 3.2.1. Full-Coverage Observation (SAR-Oriented)

Under the full-coverage observation constraint, all of the interested regions are desired be swept by the radar footprint. Assuming $R_{i}$ is the *i*th interested region and F is the whole area covered by the SAR footprint, then the full coverage constraint can be written as

(3) $$c_{1}=\left\{\begin{array}{c} 0,\;\forall P\in R,P\in F,\\ 1,\;\mathrm{otherwise}, \end{array}\right.$$

where P is one point on the ground, and $R=\underset{i}{⋃}R_{i}$ is the union of the ROIs.

#### 3.2.2. Full-Resolution Observation (SAR-oriented)

**Figure 5:**
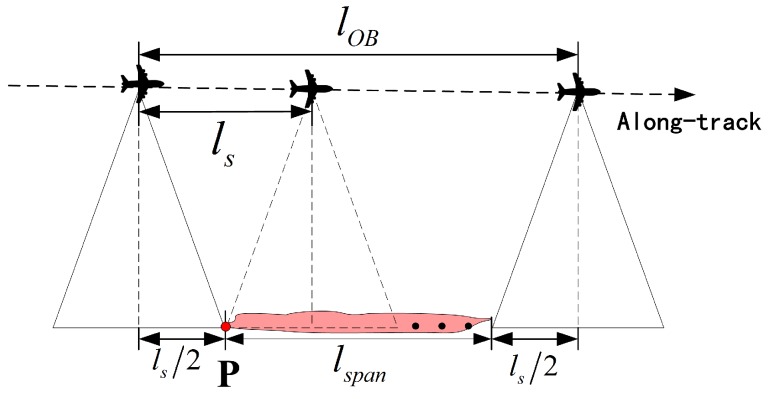
Illustration of achieving full-resolution observation of a single point P and a ROI whose span is $l_{span}$.

This constraint is induced by the unique operation characteristic of the SAR sensor. Although the curved trajectory is proved to be feasible for SAR image formation [26,57,58], a linear path is still preferred when the radar footprint passes the ROIs. This could simplify the data processing procedure, considering the lightweight UAV is not equipped with precise navigation system in general. Similar to the exposure time of the camera, its illumination duration on the targets should be long enough to achieve the full-resolution observation. To guarantee a thorough Doppler history of a target, the continuous observation duration should be longer than the synthetic aperture time [19]. As shown by Figure 5, the full resolution constraint can be given by

(4) $$c_{2}=\left\{\begin{array}{c} 0,\;\forall P\in R,L_{ob}\left(P\right)=l_{s},\\ 1,\;\mathrm{otherwise}, \end{array}\right.$$

with

(5) $$l_{s}=\frac{h}{\cos\theta_{L}}\cdot \theta_{A},$$

where $L_{ob}\left(P\right)$ is the illumination length of a point target P, $l_{s}$ is the synthetic aperture length, and $\theta_{A}$ is the azimuth beam width in radians. This constraint can be handled by analyzing the geometry features of the ROIs, which are the combinations of the point targets. As shown by Figure 2 and Figure 5, given the along-track span of one interested region, the path length corresponding to the least continuous illumination distance can be calculated by

(6) $$l_{\mathrm{ob}}=∥\vec{P_{S}P_{E}}∥=l_{\mathrm{span}}+l_{s},$$

where $l_{\mathrm{span}}$ is the ROI’s span in the along-track direction, and $∥\vec{P_{S}P_{L}|}=\vec{|P_{R}P_{E}}∥=l_{s}/2$.

#### 3.2.3. Maximum Turning Angle (UAV-Oriented)

To obey the maneuverability of the UAV, the designed path is desired to be sufficiently smooth. This means the turning angle should be within a certain limit [40,43,44]. As shown in Figure 4b, the turning angle is defined as the angle between the two adjacent directions, i.e.,

(7) $$\phi_{i}=\arccos\left(\frac{\langle \vec{p_{i-1}p_{i}},\vec{p_{i}p_{i+1}}\rangle }{∥\vec{p_{i-1}p_{i}}∥∥\vec{p_{i}p_{i+1}}∥}\right),$$

where ∥·∥ represents the Euclidean norm of one vector. Then, the maximum turning angle constraint can be given by

(8) $$c_{3}=\left\{\begin{array}{c} 0,\;\forall \phi_{i}\leq \phi_{\max},\\ 1,\;\mathrm{otherwise}, \end{array}\right.$$

where $\phi_{\max}$ is the maximum turning angle.

#### 3.2.4. Limited Map (UAV-Oriented)

Since the prior information is confined to a given space, the route should be restricted in this area to avoid the unexpected dangers [9]. This constraint function can be given by

(9) $$c_{4}=\left\{\begin{array}{c} 0,\;\forall p_{i}\in M,\\ 1,\;\mathrm{otherwise}, \end{array}\right.$$

where M is the whole C-space.

### 3.3. Objective Functions

#### 3.3.1. Minimize Path Length (UAV-Oriented)

Considering the limited flight duration of the lightweight UAV, the length of the aircraft should be minimized. In addition, the shorter path means less chance to encounter the unexpected threats. To make the evaluation more admissible in different scenarios, we normalize the actual path length by the minimum flight distance between the starting and ending positions [9]. The result is named the path length ratio (PLR), which can be calculated by

(10) $$J_{1}\left(P\right)=\sum_{i=1}^{N_{W}-1}\frac{∥\vec{p_{i}p_{i+1}}∥}{∥\vec{p_{1}p_{N_{W}}}∥}.$$

#### 3.3.2. Minimize the Risk of Kill (UAV-Oriented)

Because the surveillance mission is generally performed in the battlefield, the UAV is likely to be destroyed by military threats such as hostile missiles. Intuitively, the risk of kill (RKill) is related to the distance between the aircraft and the threat. As suggested by [43], the RKill factor can be expressed as

(11) $${\mathrm{RK}}_{k}\left(p_{i}\right)=\left\{\begin{array}{cc} \frac{{\left(R_{RK\max}^{k}\right)}^{4}}{{\left(R_{RK\max}^{k}\right)}^{4}+{\left(dM_{i}^{k}\right)}^{4}}, & \mathrm{if}\;dM_{i}^{k}\leq R_{RK\max}^{k},\\ 0, & \mathrm{otherwise}, \end{array}\right.$$

where $R_{RK\max}^{k}$ is the maximum effective distance of the *k*th missile, and $dM_{i}^{k}$ denotes the distance between the *i*th waypoint and the *k*th missile.

To acquire a better evaluation of the RKill cost, the path should be upsampled by adding a number of dividing points between the waypoints [43], as shown by the yellow dots in Figure 4. Assuming $d_{i,j}$ is the *j*th dividing points between $p_{i}$ and $p_{i+1}$, then the RKill cost of the route can be given by

(12) $$J_{2}\left(P\right)=\sum_{k=1}^{N_{M}}\left(\sum_{i=1}^{N_{W}-1}\sum_{j=1}^{N_{di}}{\mathrm{RK}}_{k}\left(d_{i,j}\right)+\sum_{i=1}^{N_{W}}{\mathrm{RK}}_{k}\left(p_{i}\right)\right),$$

where $N_{di}$ is the number of dividing points between $p_{i}$ and $p_{i+1}$, and $N_{M}$ denotes the number of missiles.

#### 3.3.3. Minimize the Risk of Radar Detection (UAV-Oriented)

Another risk confronted by the UAV is the danger to be detected by the enemy’s radar. A detailed analysis on the stealth of UAV can be found in [9]. Herein, a simplified version is adopted to describe the risk of radar detection (RRD), which can be given by [43]

(13) $${\mathrm{RD}}_{k}\left(p_{i}\right)=\left\{\begin{array}{cc} {\left(\frac{\delta_{r}^{k}}{dD_{i}^{k}}\right)}^{4}, & \mathrm{if}\;dD_{i}^{k}\leq R_{RD\max}^{k},\\ 0, & \mathrm{otherwise}, \end{array}\right.$$

where $\delta_{r}^{k}$ and $R_{RD\max}^{k}$ are intensity scale and the maximum effective radius of the *k*th radar, respectively. Similarly, the RRD cost can be evaluated by

(14) $$J_{3}\left(P\right)=\sum_{k=1}^{N_{D}}\left(\sum_{i=1}^{N_{W}-1}\sum_{j=1}^{N_{di}}{\mathrm{RD}}_{k}\left(d_{i,j}\right)+\sum_{i=1}^{N_{W}}{\mathrm{RD}}_{k}\left(p_{i}\right)\right),$$

where $N_{D}$ denotes the number of radars.

## 4. Proposed Path Planner

**Figure 6:**
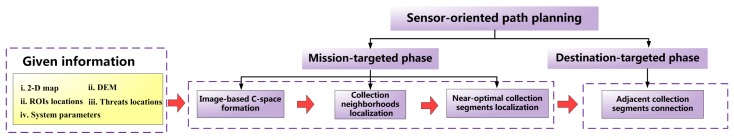
Framework of the proposed path planner.

In this section, we present an image-based path planner to achieve the full coverage of several ROIs by a single lightweight UAV SAR, the framework of which is illustrated in Figure 6.

The given information serves as the input of the path planner. First, the 2D map and the DEM could provide the information in the xy plane and the vertical dimension, respectively. They can be utilized to build the C-space and determine the flight altitude. Second, the interested and dangerous regions can be located with the prior information on their positions. Third, the swath width and location of the radar footprint can be calculated according to the SAR system parameters.

The target of conventional path planners is to design a feasible, safe, and short route from the starting position to the destination, and the aforementioned UAV-oriented factors are their main concerns. Therefore, we can call them destination-targeted planners. By comparison, the path planning problem studied in this paper puts more emphasis on the surveillance mission, i.e., covering the interested regions by the radar beam. Correspondingly, what we need is more of a mission-targeted planner. To some extent, the two types of planners conflict with each other. For example, the necessary detours to cover the interested regions are highly likely to prolong the path, which are unnecessary from the view of the destination-targeted planner. To simplify the optimization operation, we divide the problem into two subproblems that can be solved in two phases. The two phases are named the mission-targeted phase and the destination-targeted phase, respectively. First, the suitable path segments for data collection are searched near the ROIs in the mission-targeted phase, in which the SAR-oriented factors are mainly considered. The parts of route determined in the mission-targeted phase are called collection segments. After that, the remaining task is to connect the adjacent collection segments, which is implemented in the following destination-targeted phase. As shown in Figure 6, the proposed path planning method can be performed in four main steps. The overview of the main steps is summarized in Algorithm 1, the details of which will be presented in the following subsections.

**Algorithm 1** Overview of the Proposed Path Planner.
(1).*Image-based C-space formation*: Locate the interested regions in the map, then transform the map to a binary image; Merge the neighboring ROIs via the morphological operation, then detect the ROIs by the connected-component labeling technique;(2).*Collection neighborhoods localization*: Classify the ROIs via contour analysis, and then locate the boundaries of the collection neighborhoods;(3).*Near-optimal collection segments localization*: Compute the visiting sequence, search the optimal approach angle of each ROI, then search the optimal collection segment at each optimal angle;(4).*Adjacent collection segments connection*: Connect the adjacent collection segments via SSAS.


### 4.1. Image-Based C-Space Formation

In the mission-targeted phase, the main concern is the locations of the interested regions, which can be obtained with the aid of the classification techniques [59]. Herein, our path planner begins with the map on which the ROIs have already been marked.

The first step is to convert the input map to a binary image, where ones represent the ROIs, and zeros mean the background. Similar to the grid-based C-space, all the key information for the path planner (e.g., the locations and spans of the interested/dangerous regions) can be manifested by the pixel coordinates in the binary map. However, less storage and computational complexity are required compared with the color map. Hence, the planning process can be accelerated through this transformation. Then, we apply the morphological operation on the image to merge the neighboring interested regions together. The morphological operations are quite fast and simple to implement because they only involve the logical functions [60]. To keep the sizes of the ROIs unchanged, the close filter is performed here. The close filter is a combination of the basic erosion and dilation operations, i.e.,

(15) $$I_{c}\left(x,y\right)=\left[I_{0}\left(x,y\right)⊕A\left(x,y\right)\right]⊖A\left(x,y\right),$$

where $I_{0}\left(x,y\right)$ is the original binary image, $A\left(x,y\right)$ is the structuring element, and ⊕ and ⊖ denote the dilation and erosion operations, respectively. The structuring element $A\left(x,y\right)$ in Equation (15) is a shape (e.g., square, polygon, flat, circular) with much smaller size than the ROIs. The selection of the element is related to the monitored environment. Generally, a shape with the comparable size as the road, which is usually the reason for the gaps and holes in the map, can be utilized .

**Figure 7:**
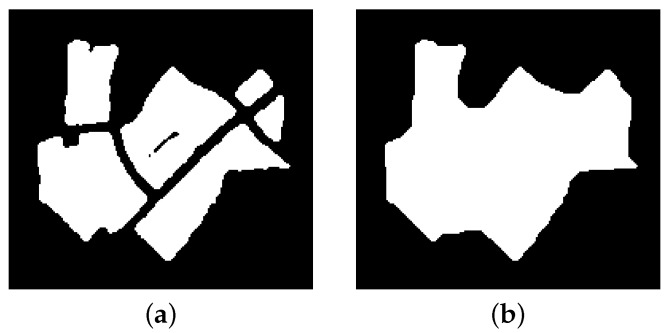
One local area with several separated ROIs. (**a**) Before the morphological operation. (**b**) After the morphological operation.

In the next, the connected components are searched using the labeling algorithm, which is a fundamental operation in pattern recognition [61]. After that, the binary image is transformed into a symbolic image, in which the label assigned to each pixel is an integer uniquely identifying the connected component to which that pixel belongs. For the path planner, the pixels with the identical label (i.e., *i* for $R_{i}$) can be seen as one ROI. One local area with several separated ROIs are shown in Figure 7a, in which six connected components can be detected. As illustrated in Figure 7b, the close filter combines several neighboring ROIs into one connected region. As for the global area, more reduction can be achieved by the morphological operation, which could decrease the computation complexity of the following procedures.

### 4.2. Collection Neighborhoods Localization

Collection neighborhood, a definition derived from [55], is the sensor location that is proper for data collection. Herein, segments near the ROI, rather than the points, are utilized to represent the data collection locations of UAV SAR. For one thing, data collection in SAR is a continuous process formed by the movement of the platform. For another, a linear path in data collection is desired. There are a number of proper collection segments around a ROI, the sum of which is its collection neighborhood. In real implementation, it is unrealistic to calculate all the collection segments. Instead, we only search the boundary segments, which can be used to represent the whole collection neighborhood. To be more specific, we firstly classify the ROIs according to their minimum rectangle contours. Based on the classification result, the boundary segments can be obtained.

#### 4.2.1. ROI Classification via Contour Analysis

**Figure 8:**
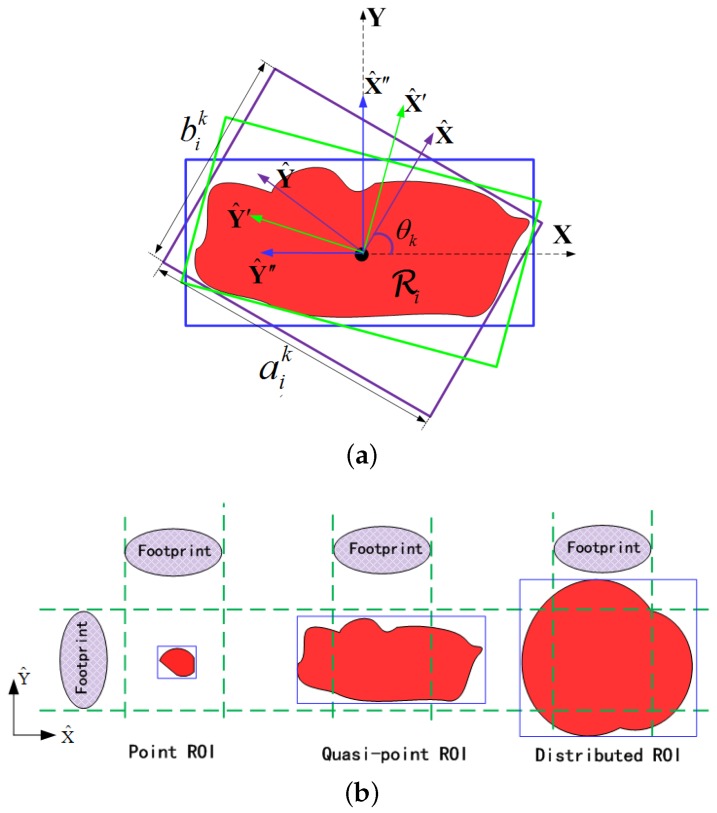
ROI classification via contour analysis. (**a**) MBRs of of an irregular-shaped ROI at different approach angles; (**b**) three categories of ROI classified by comparing the their MBR side-lengths with the SAR swath width.

Considering that the path is desired to be straight in the mission-targeted phase, we utilize the minimum bounding rectangle (MBR) [62] to describe the geometric attributes of the ROIs. The MBRs of an irregular-shaped ROI at different approach angles are demonstrated in Figure 8a, in which $a_{i}^{k}$ and $b_{i}^{k}$ are the long and the short side-lengths at the approach angle $\theta_{k}$, respectively. By comparing the side-lengths of their MBRs with the swath width, the ROIs can be classified into three categories as shown in Figure 8b, that is,

(16) $$R_{i}\in \left\{\begin{array}{cc} \mathrm{point}\;\mathrm{ROI}, & \forall \;a_{i}^{k}<l_{sw},\\ \mathrm{quasi}\text{-}\mathrm{point}\;\mathrm{ROI},\; & \exists \;b_{i}^{k}\leq l_{sw}\leq a_{i}^{k},\\ \mathrm{distributed}\;\mathrm{ROI}, & \forall \;b_{i}^{k}>l_{sw}. \end{array}\right.$$

For the point ROIs, the side-lengths at all approach angles are smaller than swath width. Hence, a point ROI can be covered in a single sweep at every approach angle. Conversely, it is impossible to cover the whole area of a distributed ROI without turns since the swath width is smaller than ROI span at all approach angles. The image-based method to classify ROI according to Equation (16) is given in Algorithm 2. It can be noted that the side-lengths of the rotated MBR are solely determined by the maximum and minimum coordinates of the ROI’s pixels (see line 6 and 8), which could only be located on the edge. Hence, the Canny edge detector [63] is performed at first to reduce the computational load (see line 2). Considering the symmetric feature of the MBR, the search scope of the approach angle is 0–90 degrees (see line 4). Additionally, the side-lengths at different angles are calculated by rotating the coordinate (see line 5). Corresponding to the counterclockwise-rotated coordinate illustrated in Figure 8a, the transformation matrix can be described as

(17) $$\begin{array}{c} T\left(\theta_{k}\right)=\left[\begin{array}{cc} \cos\theta_{k} & \sin\theta_{k}\\ -\sin\theta_{k} & \cos\theta_{k} \end{array}\right]. \end{array}$$

**Algorithm 2** ROI Classification.**Input:** A symbolic image with ROIs labeled by sequential integers. **Output:** The category of each ROI in the map. **Begin:**
1:Detect the maximum integer in the symbolic image: $N_{R}$;2:Perform Canny edge detector to the image, then search the pixel coordinates on the edge of each ROI: $E_{i}={\left[X_{i},Y_{i}\right]}^{T};$3:**for** i = 1 to $N_{R}$ **do**4:   **for** $\theta_{k}$ = 0 to 90 **do**5:      Calculate the coordinate of the edge pixels in the rotated Cartesian coordinate system: ${\hat{E}}_{i}\left(\theta_{k}\right)=T\left(\theta_{k}\right)\cdot E_{i}={\left[{\hat{X}}_{i}\left(\theta_{k}\right),{\hat{Y}}_{i}\left(\theta_{k}\right)\right]}^{T}$;6:      Calculate the two-dimensional spans of the ROI: $\Delta \hat{X}=\max\left[{\hat{X}}_{i}\left(\theta_{k}\right)\right]-\min\left[{\hat{X}}_{i}\left(\theta_{k}\right)\right]$, $\Delta \hat{Y}=\max\left[{\hat{Y}}_{i}\left(\theta_{k}\right)\right]-\min\left[{\hat{Y}}_{i}\left(\theta_{k}\right)\right]$;7:      Initialize $R_{i}$ as a distributed ROI under $\theta_{k}$;8:      Calculate the side-lengths of the MBR: $a_{i}^{k}=\max\left[\Delta \hat{X},\Delta \hat{Y}\right]$, $b_{i}^{k}=\min\;\left[\Delta \hat{X},\Delta \hat{Y}\right]$;9:      **if** $b_{i}^{k}<l_{sw}$ **then**10:         $R_{i}$ is a point ROI under $\theta_{k}$;11:         **if** $a_{i}^{k}>l_{sw}$ **then**12:            $R_{i}$ is a quasi-point ROI under $\theta_{k}$;13:         **end if**14:      **end if**15:   **end for**16:   Traverse all $\theta_{k}$ and classify $R_{i}$ according to (16).17:**end for**


#### 4.2.2. Locating the Boundary Segments

**Figure 9:**
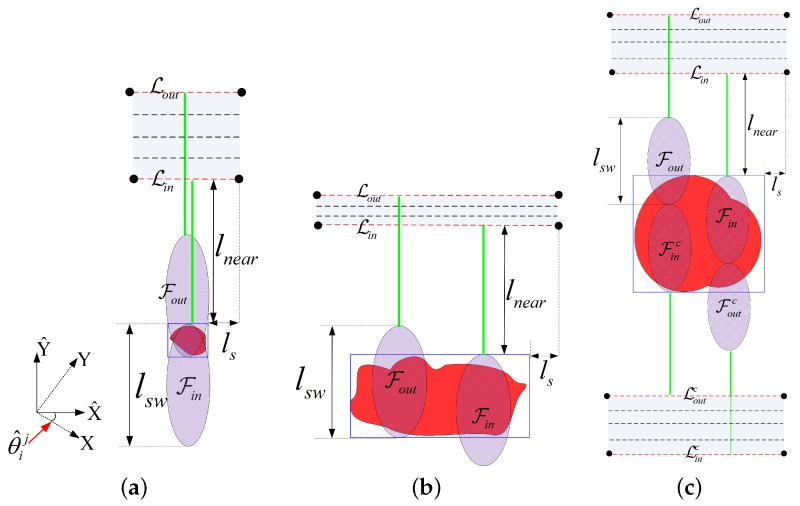
Collection segments of different ROIs. (**a**) point ROI; (**b**) quasi-point ROI; (**c**) distributed ROI.

Herein, we present two definitions to express the collection neighborhood mathematically, the illustrations of which are shown in Figure 9.

**Definition** **1.** *Given the edge pixels (i.e., $E_{i}$) of a ROI (i.e., $R_{i}$), the* feasible angles *of $R_{i}$ are a set of approach angles $\Theta_{i}=\left\{{\hat{\theta }}_{i}^{j}\right\}$, where ${\hat{\theta }}_{i}^{j}$ denotes the sweep direction to achieve full coverage with the least number of turns.*

Herein, the number of turns in the path is utilized as the indicator of the feasible approach angle for two reasons. First, turns in the path take significant time and energy [64]; second, the continuous data collection from one ROI is more preferred in conducting the reconnaissance mission.

**Definition** **2.** *Given one feasible approach angle ${\hat{\theta }}_{i}^{j}$, a* collection segment *of $R_{i}$ is one segment that satisfies the following two conditions; (1) being parallel to the x-axis of the rotated coordinate system at ${\hat{\theta }}_{i}^{j}$; and (2) being proper as a part of path to fulfill the complete coverage of $R_{i}$ with full resolution.*

Then, one collection segment can be expressed as

(18) $$L\left({\hat{\theta }}_{i}^{j},\eta \right)=\left({\hat{x}}_{l},{\hat{y}}_{l}\left(\eta \right))\to ({\hat{x}}_{r},{\hat{y}}_{r}\left(\eta \right)\right),$$

where $\left({\hat{x}}_{l},{\hat{y}}_{l}\left(\eta \right)\right)$ and $\left({\hat{x}}_{r},{\hat{y}}_{r}\left(\eta \right)\right)$ are the endpoints of the segment, η∈[0,1] is a scalar to determine the location of a segment between the boundary ones, and “→” denotes the segment between two points.

The boundary collection segments (denoted as $L_{in}$ and $L_{out}$) of the aforementioned three categories of ROIs are illustrated in Figure 9. In Figure 9, the radar footprints corresponding to the boundary collection segments are represented by $F_{in}$ and $F_{out}$, respectively. Considering that both the point and the quasi-point ROIs can be covered in a single sweep, their boundary collection segments can be obtained in the identical way, i.e., the cases when the poles of the footprint coincide with the sides of the MBR as illustrated by Figure 9a,b. In terms of the distributed category, at least a turn is required around the ROI to achieve the full coverage. For simplicity, the ROIs studied in this paper are of comparable size to the radar swath width (i.e., $\exists \;a_{i}^{k}<2l_{sw}$), so only one turn is required for the distributed ROI. The coverage of ROI with a larger area can be referred to [64,65,66]. In Figure 9c, the outside/inside footprint $F_{out}$/$F_{in}$ is the outmost/innermost one to perform the full coverage with one turn, the complementary footprint of which is denoted as $F_{out}^{c}$/$F_{in}^{c}$. Note that the collection segments at the same approach angle are parallel to each other; then, the coordinate values in Equation (18) can be written as

(19) $$\begin{array}{c} {\hat{x}}_{l}=\min\left[{\hat{X}}_{i}\left({\hat{\theta }}_{i}^{j}\right)\right]-\frac{l_{s}}{2},\\ {\hat{x}}_{r}=\max\left[{\hat{X}}_{i}\left({\hat{\theta }}_{i}^{j}\right)\right]+\frac{l_{s}}{2},\\ {\hat{y}}_{l}\left(\eta \right)={\hat{y}}_{r}\left(\eta \right)={\hat{y}}_{in}+\eta \cdot \left({\hat{y}}_{out}-{\hat{y}}_{in}\right), \end{array}$$

with

$$\begin{array}{c} {\hat{y}}_{in}=\max\left[{\hat{Y}}_{i}\left({\hat{\theta }}_{i}^{j}\right)\right]+l_{near},\\ {\hat{y}}_{out}=\min\left[{\hat{Y}}_{i}\left({\hat{\theta }}_{i}^{j}\right)\right]+\left(k_{t}+1\right)\cdot l_{sw}+l_{near}, \end{array}$$

where $k_{t}$ is the number of turns ($k_{t}=0$ for the (quasi) point ROIs, $k_{t}=1$ for the distributed ROIs), and $l_{near}=∥\vec{P_{S}P_{N}}∥$ is the ground distance between the UAV and the near-end of the footprint (see Figure 2). Here, the collection segment is extended by half of the synthetic aperture length (i.e., $l_{s}/2$) in both ends to satisfy the full-resolution observation constraint.

Given the boundary segments, any collection segment at this angle can be expressed by adjusting the value of η. Then, the collection neighborhood can be obtained by traversing all the rotating angles (0–360 degrees), which can be expressed as

(20) $$N_{i}=\underset{\theta_{i}^{j}\in \Theta_{i}}{⋃}\underset{\eta \in [0,1]}{⋃}L\left({\hat{\theta }}_{i}^{j},\eta \right).$$

Note all the coordinate values in Equations (18)–(20) are defined in the rotated coordinate system, which can be transformed to the regular coordinate system by the multiplication with $T^{-1}\left(\theta_{i}^{j}\right)$.

### 4.3. Near-Optimal Collection Segments Localization

Within the collection neighborhood, only one or two collection segments can be selected for each ROI as a part of the final path. The computational complexity to search an optimal combination of the collection segments is $O(N_{R}!\bar{N_{\theta }}N_{\eta })$, where the factorial $N_{R}!$ is induced by the visiting sequence, $\bar{N_{\theta }}$ denotes the average number of the feasible angles, and $N_{\eta }$ is the number of collection segments at each feasible angle. To reduce the computational load, we turn to search a near-optimal solution by decoupling the problem into three sub-problems: searching the optimal visiting order, searching the optimal approach angles, and searching the optimal scalars.

#### 4.3.1. Searching the Optimal Visiting Order

The main concern in choosing the visiting order is the distance between the ROIs. This a typical traveling salesman problem (TSP) that can be stated as: a salesman tries to find the shortest router to visit a set of cities under the conditions that each city is visited once [67,68,69]. Herein, the salesman is the UAV, and the cities are the ROIs. The locations of the ROIs can be represented by their centroid considering the interested regions are sparsely located in the map. There are a number of methods to solute the TSP problem, and the genetic algorithm [69] is adopted in our planner.

#### 4.3.2. Searching the Optimal Approach Angles

**Figure 10:**
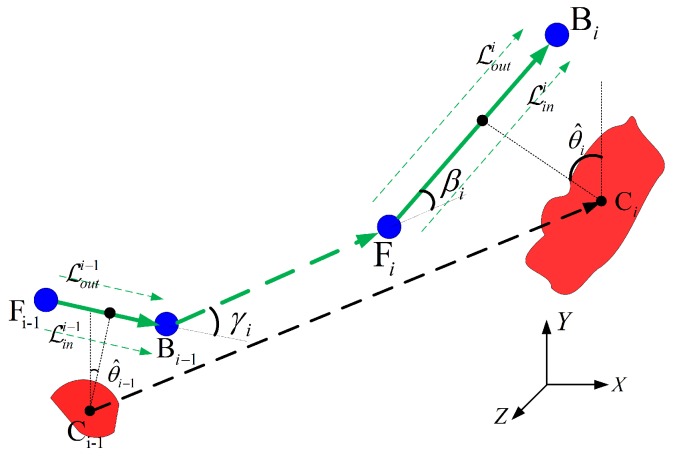
Turning angle between two adjacent collection segments.

To obtain a sufficiently smooth route, the turning angle between the adjacent collection segments (i.e., $\gamma_{i}$ pluses $\beta_{i}$ in Figure 10) should be as less as possible. In addition, the less turning angle is of advantage to save energy [64]. Moreover, the following segments connection process (see Section 4.4) can be facilitated by less orientation difference between the adjacent collection segments. This is an optimization problem that can be written as

(21) $$j_{i}^{opt}=arg min\left(\gamma_{i}+\beta_{i}\right)\;s.t.\;{\hat{\theta }}_{i}^{j_{i}^{opt}}\in \Theta_{i}.$$

**Figure 11:**
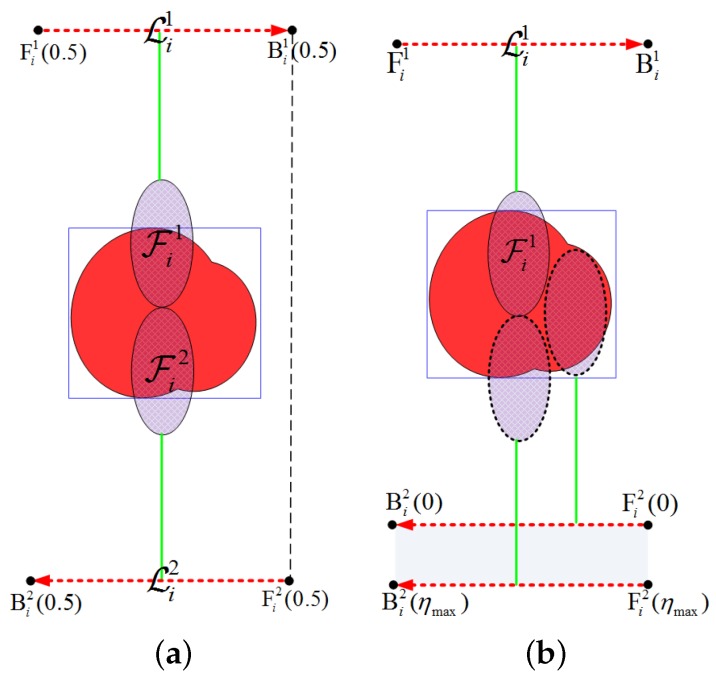
Illustration of localizing collection segments for the distributed ROI. The second segment is located on the opposite of the first one, i.e., ${\hat{\theta }}_{i,2}^{j_{i,2}^{opt}}=mod\left({\hat{\theta }}_{i,1}^{j_{i,1}^{opt}}+180,360\right)$. (**a**) $\eta_{1}=\eta_{2}=0.5$ in searching the optimal approach angle; (**b**) $\eta_{max}$ used in searching the optimal scalar of the second collection segment is restricted by the first one.

Considering the turning angle is not sensitive to η, the middle collection segment (i.e., η=0.5) is utilized in the calculation of $\gamma_{i}$ the and $\beta_{i}$ without loss of generality. The visiting order within the endpoints is determined by the viewing direction of the UAV SAR system (right viewing in Figure 10). Then, the approach direction with the minimum turning angle can be obtained by traversing all the angles in $\Theta_{i}$. Because the endpoint of the former segment (i.e., $B_{i-1}$) is involved in the angle calculation, this optimization processing is performed one by one in accordance with the visiting order. As shown in Figure 11a, two collection segments are involved in the observation of the distributed ROIs. The second collection segment (denoted as $\vec{F_{i}^{2}B_{i}^{2}}$) is located on the opposite of the first one, and its approach angle can be calculated by $mod({\hat{\theta }}_{i,1}^{j_{i,1}^{opt}}+180,360)$, where ${\hat{\theta }}_{i,1}^{j_{i,1}^{opt}}$ is the approach angle of the first segment, and mod(a,b) denotes the modulo operator.

#### 4.3.3. Searching the Optimal Scalars

Next, the optimal collection segments can be searched among those at the optimal approach angle ${\hat{\theta }}_{i}^{j_{i}^{opt}}$. Similar to Equation (21), the optimized scalar can be described as

(22) $$\begin{array}{cc} \eta_{i}^{opt}= & arg min\left(w_{J1}\cdot J_{1}\left(\vec{B_{i-1}F_{i}}\right)+w_{J2}\cdot J_{2}\left(\vec{F_{i}B_{i}}\right)+w_{J3}\cdot J_{3}\left(\vec{F_{i}B_{i}}\right)\right),\\ & s.t.\;\;\eta_{i}^{opt}\in \left[0,\eta_{\max}\right],\;\vec{F_{i}B_{i}}\in \left\{L\left({\hat{\theta }}_{i}^{j_{i}^{opt}},\eta \right)\right\}, \end{array}$$

where $w_{J1}$, $w_{J2}$, and $w_{J3}$ are the weighting coefficients. Because the segments at the same approach angle have the same length, only the length of $\vec{B_{i-1}F_{i}}$ is considered here. In addition, we search the best η one by one in accordance with the visiting order. The value of $\eta_{\max}$ is 1 in most of the cases except for the second collection segment of the distributed ROI. As shown in Figure 11b, $\eta_{\max}$ of the second collection segment is restricted by first one, and $\eta_{\max}=1-\eta_{i}^{opt1}$, where $\eta_{i}^{opt1}$ is the predetermined optimal scalar corresponding to the first collection segment of the *i*th ROI.

### 4.4. Adjacent Collection Segments Connection

After the mission-targeted phase, the remaining task is to connect the adjacent collection segments. This is a set of independent destination-targeted route planning process, which can be solved in parallel.

#### 4.4.1. Sampling-Based Search Structure

In the conventional SAS algorithm [39], the search operation is implemented in a precalculated map cost array. However, it is unnecessary to compute a global map cost array since each sub-path is only designed in a local area of the map. In addition, it is time-consuming to update the MC array when the environment changes [44]. To avoid the explicit construction of a search map, the sampling strategy [70] is implemented here, in which the surrounding environment is probed by a growing search tree.

**Figure 12:**
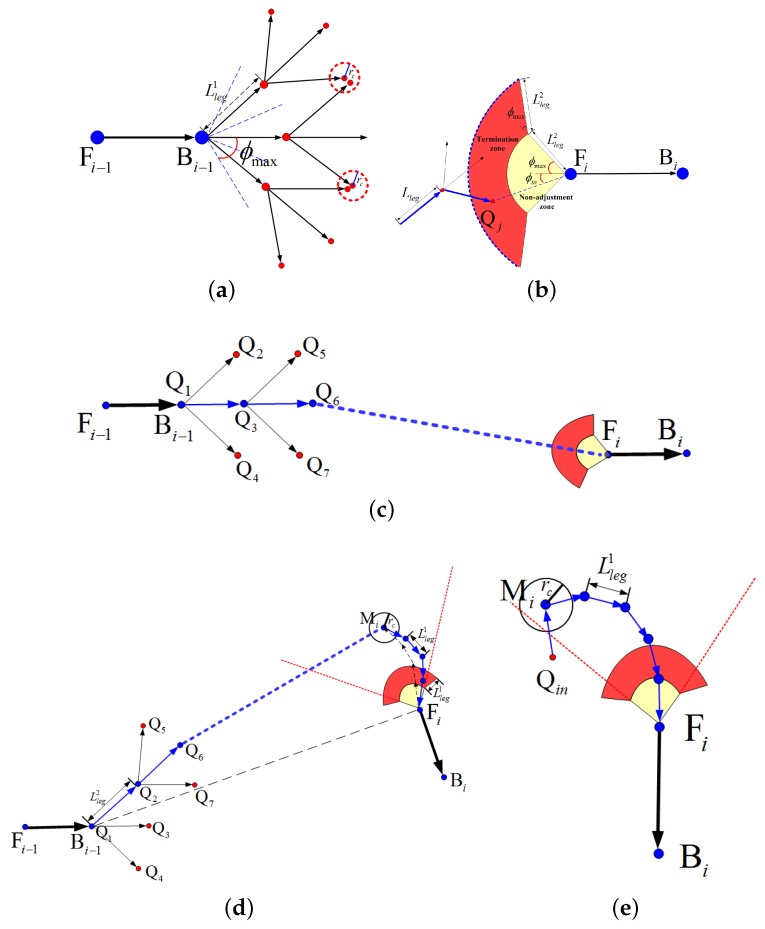
Illustration of the proposed SSAS algorithm. (**a**) the search tree utilized to sample the C-space; (**b**) ring-shaped termination zone used to achieve a smooth approaching to the ending collection segment; (**c**) snapshot of SSAS with small orientation difference between the adjacent collection segments; (**d**) snapshot of SSAS with large orientation difference between the adjacent collection segments; and (**e**) extreme case requiring a U-turn.

The tree-like sampling structure is illustrated in Figure 12a. At the beginning, an initial fan-tail is extended from the starting waypoint along the sweep orientation, the span of which is $2\phi_{max}$ to cover all feasible turning angles. If no initial heading is provided (e.g., at the very beginning of the whole path), the fan-tail is 360 degrees around the waypoint. Then, the fan-tail is the divided into several equal sectors, the number of which will determine the precision and speed of the search operation. As suggested by [39], three to five sectors are sufficient for the real-time application. The branches are located in the centers of the sectors, the length of which are the leg lengths (denoted as $L_{leg}$ in Figure 12a).

Each tree node serves as a sample of the C-space, which is evaluated by the objective costs of a route passing through the node. The evaluation path is composed of two parts: the first is the actual component from the starting waypoint to the node, and the second is the heuristic component from the node to the ending waypoint. Then, the cost of a tree node $Q_{j}$ for a specific objective function $J_{k}$ can be written as

(23) $$J_{k}\left(Q_{j}\right)=g_{k}(Q_{j})+\alpha_{k}\cdot J_{k}\left(\vec{Q_{j}F_{i}}\right),$$

with

$$g_{k}\left(Q_{j}\right)=J_{k}\left(B_{i-1}⇀Q_{j}\right),$$

where $B_{i-1}⇀Q_{j}$ denotes the path from the starting waypoint $B_{i-1}$ to the node $Q_{j}$, and $g_{k}\left(Q_{j}\right)$ is the actual cost along this path. Then, we can obtain

(24) $$\left\{\begin{array}{c} g_{k}\left(Q_{j}\right)=g_{k}\left(Q_{j}^{p}\right)+J_{k}\left(\vec{Q_{j}^{p}Q_{j}}\right),\\ g_{k}\left(Q_{j}^{c}\right)=g_{k}\left(Q_{j}\right)+J_{k}\left(\vec{Q_{j}Q_{j}^{c}}\right), \end{array}\right.$$

where $Q_{j}^{p}$ and $Q_{j}^{c}$ are the parent and child nodes of $Q_{j}$, respectively. The second term in Equation (23) is the heuristic to estimate the cost from $Q_{j}$ to the ending waypoint, where $\alpha_{k}$ is the heuristic weight to balance the actual and estimated costs. To obtain a near-optimal route, the heuristic must be admissible, i.e., the combined cost to the goal should never be overestimated. The weight is usually set to 1 in searching the shortest route (i.e., the PLR factor), considering the distance factor would never be overestimated in this case (a straight line is physically the smallest possible distance between any two points). As for the two threat factors, less weights are preferred to avoid overestimating the costs.

Without the restriction of a predefined search map, the sampling strategy provides high freedom for the path planner. One disadvantage of this sampling-based planner is the huge number of candidate nodes in the search process, especially when the searcher goes to the deep layer of the search tree. To reduce the computational load, one branch will be ignored if its nearby area has already been reached. As shown in Figure 12a, this area is defined as a circle around the node, the radius of which is $r_{c}$. In addition, cost comparison will be performed between the newcomer and the former sampler of this area. If the new node costs less, it will replace the old one as the sample of this area.

#### 4.4.2. Selection and Extension of the Best Node

In the destination-targeted phase, the aforementioned three UAV-oriented objectives are involved, i.e., minimizing the costs of the PLR, RKill, and RRD factors. Similar to Equation (22), the weighted sum model (WSM) is used to combine the three objectives, i.e.,

(25) $$J\left(Q_{j}\right)=\sum_{k=1}^{3}w_{k}J_{k}\left(Q_{j}\right),$$

where $J_{1}\left(Q_{j}\right)$, $J_{2}\left(Q_{j}\right)$, $J_{3}\left(Q_{j}\right)$ are costs of PLR, RKill and RRD, respectively. Coefficients $w_{1}$, $w_{2}$, $w_{3}$ are their corresponding weight coefficients.

The proposed SSAS is summarized in Algorithm 3. In SSAS, the obtained nodes are stored in a priority queue named open set. At each iteration, the node with the least cost is selected from the open set to generate the new child nodes. After the extension, the parent node is moved into another queue called closed set (see line 4). The new nodes would be added to the open set if their neighborhoods have never been reached (see line 17). The loop continues until the predefined termination condition is satisfied or the open set becomes empty. A feasible route is obtained if the search process is ended by the predefined termination condition. Then, the route can be obtained by tracing back the predecessors from the termination node until the starting waypoint is reached (see line 6). The termination conditions of SSAS will be presented in the following.

**Algorithm 3** Sampling-based Sparse A* Search Algorithm.**Input:** The starting position, the destination position, the search leg length, and the termination condition **Output:** Coordinates of the waypoints **Begin:**
1:Empty the open set and the close set;2:Set the starting waypoint as the first node $Q_{1}$, add $Q_{1}$ to the open set ($J(Q_{1})=0$), and initialize the number of the explored node $N_{j}=1$;3:**repeat**4:   Select the best node $Q_{m}$ from the open set, move $Q_{m}$ from the open set to the close set;5:   **if** the termination condition is satisfied at $Q_{m}$ **then**6:      Terminate the iteration, then trace back the path up the search tree from $Q_{m}$ until the starting waypoint is reached (**return** the traced path);7:   **end if**8:   Expand the search tree from $Q_{m}$, and obtain the children node set $\left\{Q_{m}^{c}\right\}$;9:   **for** each child node in $\left\{Q_{m}^{c}\right\}$ **do**10:      Calculate its distances from the other reached nodes ${\left\{Q_{j}\right\}}_{j=1,2,\ldots ,N_{j}}$, and select the nearest node $Q_{n}$ from $\left\{Q_{j}\right\}$ with minimum distance $r_{min}$;11:      **if** $r_{min}<r_{c}$ **then**12:            This is a node that has been reached before;13:            **if** $J\left(Q_{m}^{c}\right)<J\left(Q_{n}\right)$ **then**14:               Replace $Q_{n}$ by $Q_{m}^{c}$ ;15:            **end if**16:      **else**17:            A new node is obtained, $N_{j}=N_{j}+1$, and add $Q_{m}^{c}$ to the open set as $Q_{N_{j}}$;18:      **end if**19:   **end for**20:**until** the open set is empty (**return** failure)


#### 4.4.3. Termination Conditions

To obtain a smooth approaching at the ending collection segment, a fan-tail is added to the ending segment as shown in Figure 12b. The UAV should adjust its heading direction before entering the yellow sector, which is called the non-adjustment zone. The side-length of the non-adjustment zone is the minimum distance for the UAV to change its heading direction [39]. Similar to the bucket-shaped region in [39], a ring-shaped termination zone for heading adjustment is designed here, which is marked in red in Figure 12b. Constrained by the maximum turning angle, the UAV should enter the termination zone from its outer margin (see the blue dotted line). Hence, the search process will terminate if and only if we acquire a tree node $Q_{j}$ that satisfies the following two conditions:

(26) $$\left\{\begin{array}{c} L_{leg}\leq ∥\vec{Q_{j}F_{i}}∥\leq 2L_{leg},\\ \phi_{in}\leq 2\phi_{max}, \end{array}\right.$$

where $\phi_{in}$ is the angle between $\vec{B_{i}F_{i}}$ and $\vec{F_{i}Q_{j}}$. As for the ending position without restriction on the approach direction (e.g., the final destination or an intermediate point), only the first condition in Equation (26) is required.

#### 4.4.4. Bidirectional Search Strategy

The heuristic term in the evaluation function (see Equation (23)) is computed by a direct connection between the node and the destination, in which the approach angle is not accounted for. When the orientation difference between the adjacent vectors is small, the search tree is able to approach the destination in the proper direction under the guidance of the heuristic as shown in Figure 12c. However, this heuristic cannot estimate the cost well if the orientation difference becomes large. One instance is shown in Figure 12d, where the vector $\vec{F_{i}B_{i-1}}$ is out of the scope of the termination ring. In this case, the heuristic term is inaccurate, and the tree will be induced to the inappropriate approach direction. This is one reason why the turning angle between two adjacent collection segments is desired to be minimized in selecting the collection segments (see Equation (21)).

To lead the search tree to the proper approach direction, a bidirectional search strategy is utilized in our sub-path planner. As illustrated by the snapshot in Figure 12d, we implement SSAS three times in three stages. The leg lengths in the first and the third stage are $L_{leg}^{1}$, which are smaller than the that (denoted as $L_{leg}^{2}$) used in the second stage. In the first stage, the sub-path planner begins from $F_{i}$ with a small leg length $L_{leg}^{1}$, aiming at $B_{i-1}$. In this inverse search process, the selected node must be within the scope of the termination ring as marked by red dotted line in Figure 12d. Assuming $Q_{k}$ is one selected node in the inverse search process, then the angle between $\vec{B_{i}F_{i}}$ and $\vec{F_{i}Q_{k}}$ must be less than $\phi_{max}$. In addition, the inverse search process is set to stop near the non-adjustment zone, which can be achieved by restricting the number of extension times $N_{ex}$ in the search process. Then, the ending node $M_{i}$ would serve as the destination of the second search stage, which begins from $B_{i-1}$. In this stage, the leg length can become comparatively large. Once the search tree enters the neighborhood of $M_{i}$, it comes to the third search stage. At the third stage, the destination is $F_{i}$. Figure 12e shows the extreme case in which the route designed in the second stage approaches $M_{i}$ from the opposite direction of $\vec{F_{i}B_{i}}$. In this case, a turn of 180${}^{\circ }$ (U-turn) is required in the third stage. To allow possible turning angles, the extension times $N_{ex}$ in the first stage should be large enough, i.e.,

(27) $$N_{ex}\geq \left\lceil \frac{180^{\circ }}{\Delta \phi_{max}}\right\rceil ,$$

where ⌈·⌉ is the ceiling operation. In terms of the sub-path involving only one vector (e.g, the sub-path aiming at the final destination), the tree would grow from the vector to the point, which means only the second stage is involved.

## 5. Experimental Results

### 5.1. Scenario Description and Pretreatment

**Figure 13:**
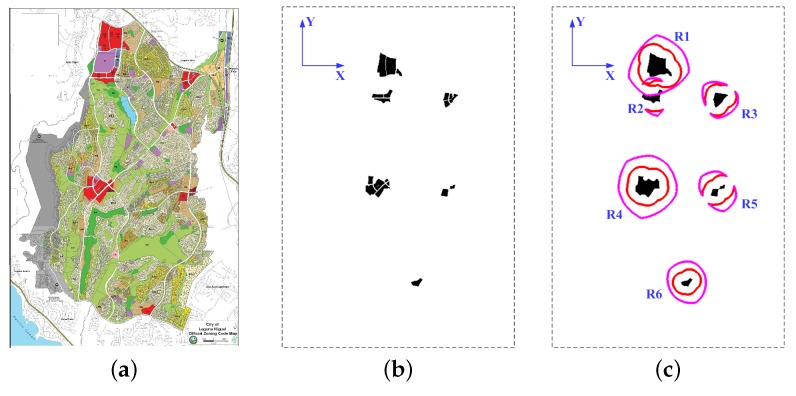
Pretreatment of the input map. (**a**) the input map image: the official zoning map of the city of Laguna Niguel; (**b**) binary image after ROI detection; (**c**) symbolic image after the image-based pretreatment operations.

There are no widely accepted benchmark problems in the field of UAV path planning, so the simulated environment is generally used in the existing literature [39,40,41,42,43,44]. In the real implementation, the path planner is usually applied in a map on which the interested regions have already been marked. Considering the ROIs can be located with the aid of the classification technique, we utilized a classification map as the input of the path planner in the simulation experiments. As shown in Figure 13a, the selected classification image is the official zoning map of the city of Laguna Niguel [71]. The input zoning map consists of 1754 by 1240 pixels, and its spatial resolution is 5.4 m per pixel. The whole map is classified to 17 regions according to the land-use. In the experiment, we selected the community commercial districts (marked in red) as the ROIs. For one thing, they are distributed sparsely in the map. For another, the sizes of these districts are various, including the three kinds of ROIs mentioned in Section 4.2. The selected districts were extracted by detecting the red areas in the map, in which the threshold was set as 0.18. The threshold is larger than zero in order to discard the near-red (e.g., pink and purple) regions. Then, the image was converted to a binary image as shown in Figure 13b. In addition, the image size was expanded to 2200 by 1500 pixels for a larger map limit to design test cases. It can be noted that there exist many gaps and holes within the detected ROIs. Hence, a number of independent ROIs would be detected if the connected component labeling operation is implemented directly on this binary image. As aforementioned, the close operation was implemented to merge the neighboring ROIs. The morphological structuring element is a disk with the radius being 15 pixels (81 m). After the morphological operation, there is merely a slight change in the size of each interested region, but the number of the detected connected components is reduced from 19 to 6. Then, each connected component is recognized as an independent ROI, the label of which is shown in Figure 13c.

The radar parameters in the experiments are shown in Table 1. Without loss of generality, the UAV SAR operates in the right side-looking stripmap mode. According to the DEM information [72], the maximum elevation of this area is 408.91 m. To avoid the collision against the terrain, the flight altitude is set as 500 m, which is a normal altitude for the lightweight UAVs [4]. As illustrated in Table 1, the swath width of the radar system is only 577.35 m. In this case, the radar footprint cannot cover some large ROIs (e.g., R1 in Figure 13c) in a single sweep. By comparing the side lengths of their MBRs with the swath width, the ROIs were classified to three categories, and the classification result is shown in Table 2. In the next, the boundary collection segments can be searched. For an explicit illustration, only the midpoints of the boundary collection segments are plotted in Figure 13c, and they form an encirclement around each interested region. Note the gaps in the encirclements of the quasi-point ROIs, which represent the infeasible approach angles.

### 5.2. Performance of the Proposed Path Planner

In the following experiments, four test cases based on the pretreated scenario are designed to evaluate the performance of the proposed path planner. As shown in Table 3, six missiles and three radars are located in the map. The effective radius of missile and radar are set as 800 m and 600 m, respectively. Moreover, the threats are located in the same places in the four test cases. The differences among the four test cases lie in the locations of the starting and ending positions, which lead to distinctive routes designed in the same scenario.

As for the parameters of the path planner, the fine leg length $L_{leg}^{1}$ is set as 100 m. The coarse leg length $L_{leg}^{2}$ is $∥\vec{B_{i-1}F_{i}}∥/8$. Considering the UAV cannot adjust its heading direction too abruptly [39], the minimum value of $L_{leg}^{2}$ is set as 100 m (i.e., if $∥\vec{B_{i-1}F_{i}}∥/8<100\;$m, $L_{leg}^{2}=100\;$m). The maximum turning angle is $30^{\circ }$ [44], and five sectors are utilized in the search tree [39]. To achieve a uniform effect of the PLR, RKill, and RRD factors, the weights for the three terms in Equations (22) and (25) are all set as 1 [45]. In addition, the distance between the dividing points are set as 100 m in calculating the route costs.

**Figure 14:**
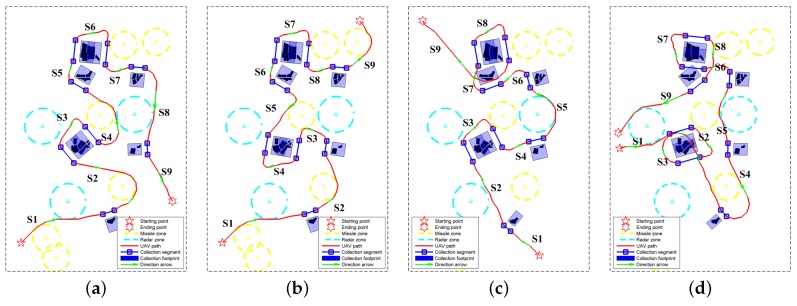
Paths designed by the proposed path planners. (**a**) test case 1; (**b**) test case 2; (**c**) test case 3; (**d**) test case 4.

The routes designed by the proposed path planner are shown in the first row of Figure 14. In Figure 14, the starting and ending points are marked by the pentagram and the hexagram, respectively. The dangerous areas are within the yellow and cyan circles, which represent the effective regions of the missiles and radars, respectively. As mentioned in Section 4, the collection segments are firstly determined in the mission-targeted phase, and these segments are marked by blue lines with two square markers at their ends. According to geometry model shown in Figure 2, the areas illuminated by the radar beam when UAV flies along the collection segments can be calculated. Because there is no turn in the data collection segments, we label the collection footprints by blue rectangles in Figure 14. Moreover, the sub-paths designed by SSAS are plotted by the red curves, which are formed by connecting the waypoints. For a clear illustration, green arrows are used to show the path directions in Figure 14.

As shown in Figure 13c, there are six labeled ROIs after the morphological operation. Since R1 and R4 belong to the distributed ROIs involving two sweeps, eight collection segments are obtained in the mission-targeted phase. As illustrated by Figure 14a–d, all the ROIs are completely covered by the collection footprints, which are coupled with the collection segments. Corresponding to the eight collection segments, nine sub-paths are designed by the proposed SSAS algorithm. The sub-paths are numbered according to the access sequence in Figure 14 (i.e., S*k* denotes the *k*th sub-path). Figure 14a shows the designed route of test case 1, in which the starting and ending points are both located in the south of the map. To cover the interested regions in the north, the UAV goes up after visiting R6. Hence, the path length is much longer than the direct connection between the initial point and the destination. Thanks to the limit of the maximum turning angle, the designed path is quite smooth. In addition, all the dangerous regions are avoided in the desinged path: Note the path planner successfully finds the corridors between the dangerous circles in s1 and s4. Next, the ending point is moved to the upper part of the map in test case 2. It can be observed from Figure 14b that the path planner changes the visiting order due to the change of destination. Correspondingly, different collection segments are chosen for R4 and R5. Again, a smooth path is obtained by SSAS as shown in Figure 14b. Figure 14c plots the designed path of test case 3. Connecting the collection segments in case 3 is quite simple, because only the fifth sub-path (i.e., s5) are blocked by the radar circle. Lastly, we place the destination near the starting position in test case 4. Because the starting and ending points are located in the center along the north-south direction, the TSP solver separates the ROIs in two parts, and the regions in the south are visited first as illustrated in Figure 14d. Along the designed route, the UAV could return to the original place after sweeping all the interested regions via the SAR footprint.

**Figure 15:**
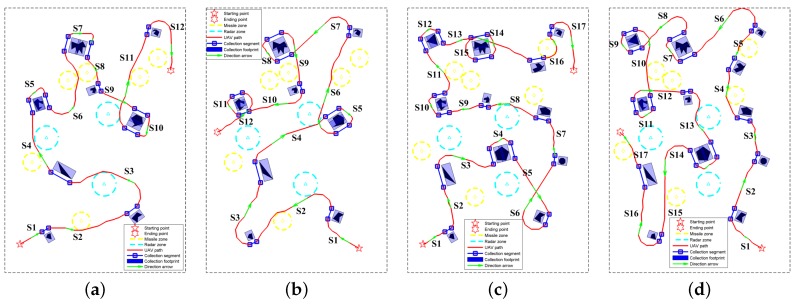
Designed path with more ROIs. (**a**) test case 5; (**b**) test case 6; (**c**) test case 7; (**d**) test case 8.

In addition, we tested the proposed method in the environment with more ROIs. As shown in Figure 15, the proposed planner succeeded in designing routes with all the ROIs being covered by the radar footprint in the four test cases. The computational time of the main steps the proposed method in the aforementioned eight test cases is listed in Table 4. All the results were obtained via MATLAB 8.3 software (The Mathworks Inc., Natick, MA, USA) on a laptop with Intel i7-6820HQ @2.70 GHz CPU. It can be noted that the time it took to locate the collection neighborhoods (Step 2) and the near-optimal collection segments (Step 3) is positively related to the number of ROIs. The more ROIs are included in the map, the more time is required. When it comes to the fourth step, the positive correlation is not satisfied in some cases. For example, the time the planner took in case 4 is much more than (3–6 times) that in case 1–3 although the same number of ROIs are included in the environment. This is mainly because the orientation difference in S3 of case 4 is pretty large, and a huge turn is required. In the search operation, the search tree grew to the opposite direction at first, and it took a lot of time for the planner to find a way towards the destination of this sub-path. The same problem was also encountered in case 6 (S12) and case 7 (S15), which prolonged the path-designing time.

Next, three sub-paths (S1, S4, and S8 in test case 1) are selected to test the influence of the nearby radius $r_{c}$ and the heuristic weights $\alpha_{k}$ on the cost and computational time of the designed path. As shown in Figure 14a, different circumstances are confronted in designing the three sub-paths. In S8, the adjacent collection segments are slightly blocked by a radar zone, which can be avoided easily. When it comes to S1 and S4, circumstances become more complicated with more dangerous zones between the adjacent collection segments.

**Figure 16:**
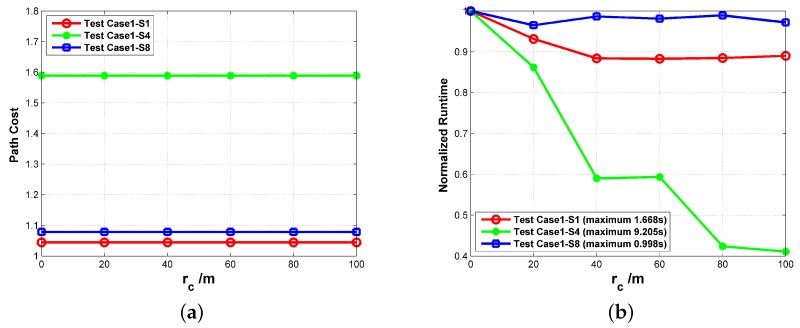
Influence of nearby radius $r_{c}$ on the designed path. (**a**) influence on path cost; (**b**) influence on computational time.

The value $r_{c}$ should not be too large in the experiment. With a large $r_{c}$, the open set in Algorithm 3 tends to become empty easily when a number of nodes are seen as the identical sampler of the C-space. Considering the fine leg length is 100 m, the maximum value of $r_{c}$ in the experiment was set as 100 m. Within this interval, the value of $r_{c}$ has no influence on the path cost as shown in Figure 16a. On the other hand, the increase of rc could induce more reduction of the computational time within this interval. In Figure 16b, the computational time is normalized to their maximum value to shown the variation trend more clearly. It can be noted that the reduction is greater in the more complicated circumstances (S4 > S1 > S8). This is mainly because the search tree grew deeper when more complicated circumstances were confronted, and more nearby nodes were eliminated in the search process. All the routes in Figure 14 and Figure 15 were obtained with rc being 50 m.

**Figure 17:**
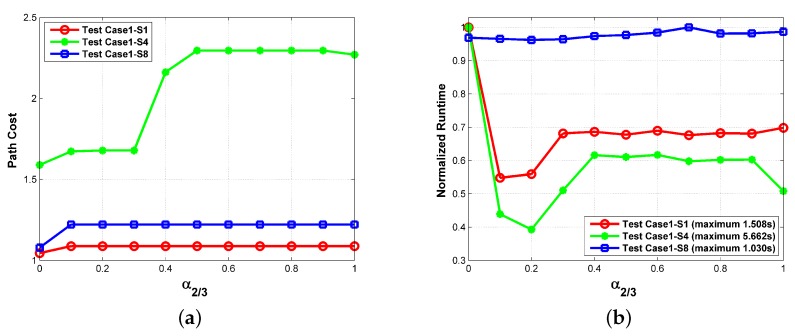
Influence of heuristic weights $\alpha_{k}$ on the designed path. (**a**) influence on path cost; (**b**) influence on computational time.

**Figure 18:**
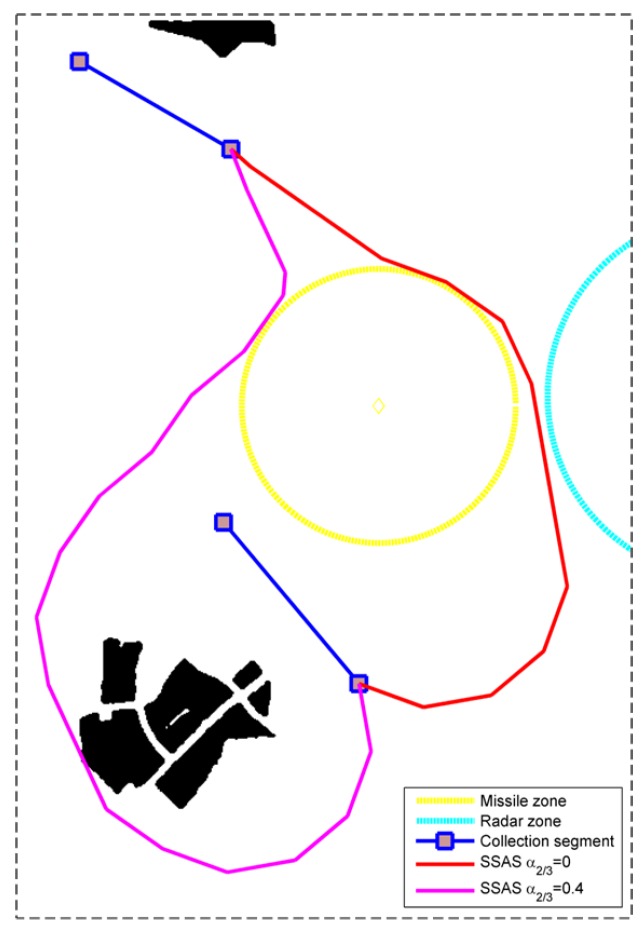
Designed sub-paths with different $\alpha_{k}$ in test case 1-S4.

In terms of heuristic weights, their values are generally less than 1 to avoid the overestimation of the path cost. Herein, we mainly concentrated on the influence on the threat-related heuristic weights. Because the uniform weights are adopted in calculating the path cost (i.e., $w_{2}=w_{3}=1$ in Equation (25)), the two threat-related heuristic weights are set as the same value (i.e., $\alpha_{2}=\alpha_{3}$) with $\alpha_{1}$ being 1 in this experiment. We can see from Figure 17 that the influence of $\alpha_{2/3}$ also varies in different circumstances. For the simplest circumstance in S8, the variation of $\alpha_{2/3}$ can be neglected. As for S1 and S4, the minimum path cost is obtained when $\alpha_{2/3}=0$ at the expense of the longest runtime. Moreover, the increase of $\alpha_{2/3}$ leads to the prolonged runtime. When $\alpha_{2/3}>0$ , the path cost does not change with the heuristic weights for S1. As for S4, there is a step change of path cost at $\alpha_{2/3}=0.4$, which comes from the change of detour direction as shown in Figure 18. To minimize the path cost, all the routes in Figure 14 and Figure 15 were obtained with $\alpha_{1}=1,\alpha_{2/3}=0$. If the computational efficiency is more emphasized, a larger heuristic weight can be adopted.

### 5.3. Performance Comparison

#### 5.3.1. Compared with the Conventional Zigzag Path Planner

**Figure 19:**
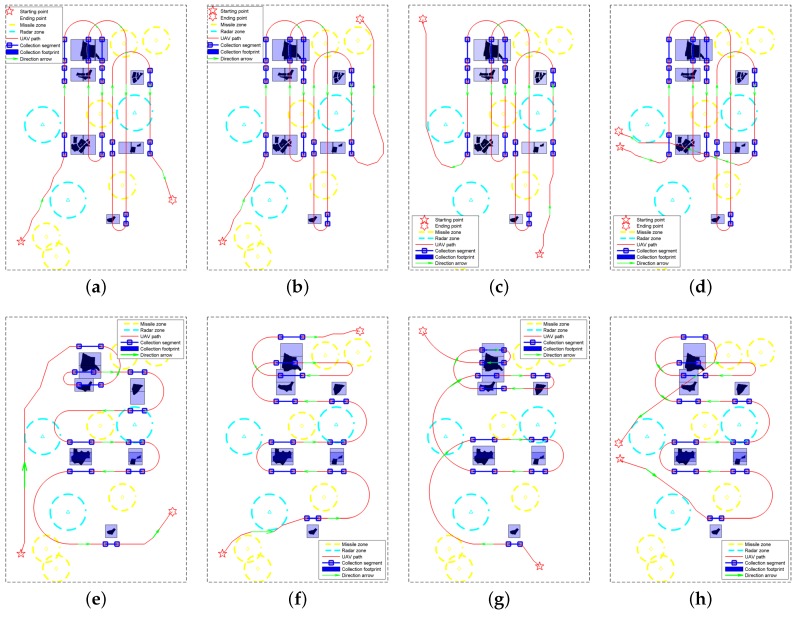
Vertical (top) and horizontal (bottom) zigzag paths. (**a,e**) test case 1; (**b**,**f**) test case 2; (**c**,**g**) test case 3; (**d**,**h**) test case 4.

Route in the zigzag pattern has been widely used as the solution to the coverage path planning problem [34]. Establishing the problem addressed here as a coverage path planning problem, the vertical and horizontal zigzag paths designed for the four cases are plotted in Figure 19. In designing the zigzag paths, all interested regions are combined as a whole, and a bounding rectangle is used to encircle the whole area. Then, the UAV is designed to move along the edges of the rectangle. As shown in Figure 19, six and seven turns are required for the vertical and horizontal zigzag paths, respectively. In designing the paths, the unnecessary routes can be avoided according to the locations of the ROIs. Moreover, the sweep directions are determined by the locations of the starting position and the destination.

Herein, we mainly compare the two path planners in the length factor. The route lengths (RLs) in Figure 14 and Figure 19 are listed in Table 5. We can see that the proposed path planner reduces the route lengths dramatically in contrast with the conventional zigzag route planner. The route lengths of the vertical zigzag paths in the four test cases are all more than 50 km, and the values of the horizontal zigzag paths are all more than 40 km. Mainly thanks to the TSP solver, the proposed method reduces the distances to around 30 km in all test cases. This reduction make it even possible to complete the reconnaissance mission by a single civil UAV. Taking the DJI phantom 4 UAV as an example, its maximum flight time is 28 min at a speed of 20 m/s. That is to say, the maximum flight distance is 33,600 m, which is beyond the path lengths designed by the proposed path planner in the four test cases.

**Figure 20:**
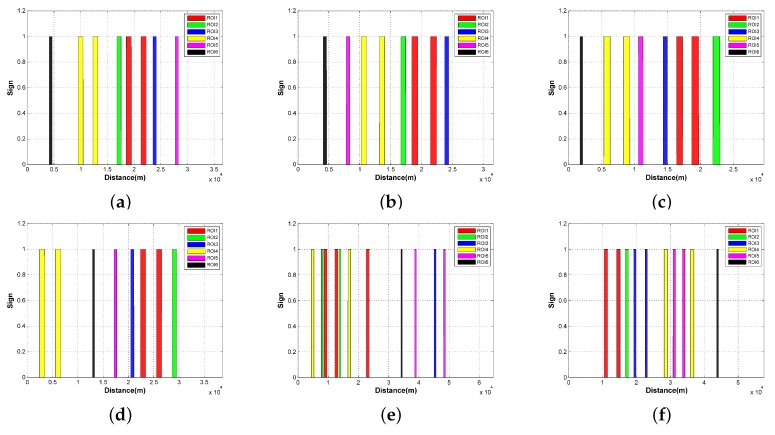
Operating functions of the designed paths. (**a**) path in Figure 14a; (**b**) path in Figure 14b; (**c**) path in Figure 14c; (**d**) path in Figure 14d; (**e**) zigzag path in Figure 19a; (**f**) zigzag path in Figure 19e.

Another performance indicator for comparison is the path length for data collection. Since only the information collected from the ROIs matters, the radar system does not need to work all the time. Once the flight path is determined, the operating intervals of the SAR system can be set by referring to the locations of the interested regions. For one thing, this is of help to save battery life. For another, the radio silence has the advantage of reducing UAVs’ vulnerability in conflict areas. The collection lengths (CLs) of the four test cases are listed in Table 5. The reductions in operation durations mainly come from the appropriate selection of approach angles around the ROIs. Furthermore, we divide the collection length of each path by its whole path distance, the outcome of which is defined as the path’s duty cycle (DC) in operating the reconnaissance mission. The increased duty cycles indicate a more efficient reconnaissance can be achieved by the proposed path planner. For a better illustration, we plot the operating functions in Figure 20. The function values equal 1 in the operating intervals, otherwise they are 0. As for the zigzag pattern, only the operating functions in test case 1 are shown in Figure 20e,f. Compared to the paths designed by the proposed planner, the two zigzag routes have more sparse distribution of the operating intervals, which are another indication of their low duty cycle. From Figure 20, another benefit of the proposed path planner can be seen, i.e., a faster and more continuous data collection of one ROI can be achieved. Take R4 (marked in yellow in Figure 20) as an example: although the vertical zigzag path also sweeps the region in two times, the path interval between the two visits are more than 10 km (Figure 20e). By contrast, this value is less than 5 km in Figure 20a–d. Furthermore, there is no visit to other ROIs between the two visits in our path. In the real reconnaissance mission, the visit order sometimes needs to be adjusted since the environment is changing. The faster and more continuous coverage of the former ROI is advantageous to adjust the following visit sequence.

#### 5.3.2. Compared with the Other Optimal Path Planning Algorithms

As aforementioned, the optimal solution to the path planning problem is NP-complete, and the stochastic methods are proved to be effective in solving NP-hard problems. In the following experiments, three state-of-the-art stochastic path planning methods were implemented as the comparison with the proposed SSAS algorithm. The first planner [9] is based on the genetic algorithm (GA). It initializes the candidate waypoints in a Polar coordinate system and evolves them in the Cartesian coordinate system. In addition, the binary tournament method is used to select qualified elements to breed the next generation. In the second planner, the waypoints are evolved separately by a differential evolution (DE) algorithm named JADE [43]. The third path planning algorithm for comparison is the PSO algorithm [54]. Considering that the three compared algorithms are all destination-targeted, we only compare their performance in designing sub-path (i.e., Step 4 in Figure 6). The evaluation criteria used in the three compared algorithms can be described as: (1) the sub-path with less constraints being violated (i.e., $c_{3}+c_{4}$ in Equation (2)) is better; (2) with the same number of constraints being violated, the sub-path that violates the maximum turning angle constraint less times is better; (3) if two sub-paths are equally evaluated under the aforementioned two criteria, the one with less path cost (i.e., Equation (25)) is better. In the experiments, the three reference algorithms also codify the path as a sequence of linear segments [43]. In addition, they terminate after 100 generations. In all, we conducted each path planning methods 100 times to evaluate their statistical performance. The parameters used in the three planners are listed in Table 6. The definition of these parameters can be found in the corresponding references. The two sub-paths in this comparative experiment are both selected from test case 1 (see Figure 14a). The first one is s1, which is a connection between a point (i.e., the starting position) and one vector (i.e., the first collection segment). The second sub-path for comparison is s4. Compared with s1, s4 is a connection between two vectors. Furthermore, the angle between the two vectors is almost 180 degrees, making it complicated to design a feasible path under the maximum turning angle limit.

**Figure 21:**
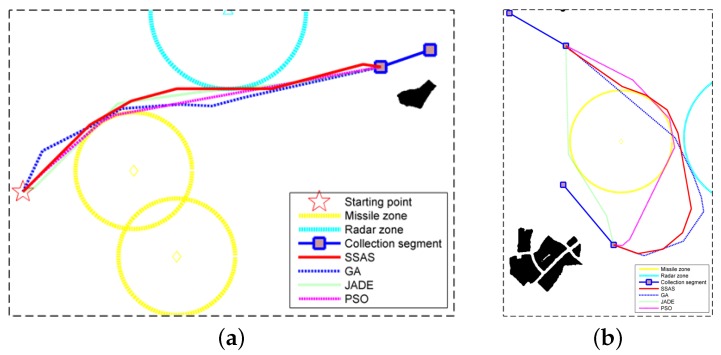
Sub-paths designed by different path planning algorithms. (**a**) test case 1-s1; (**b**) test case 1-s4.

**Figure 22:**
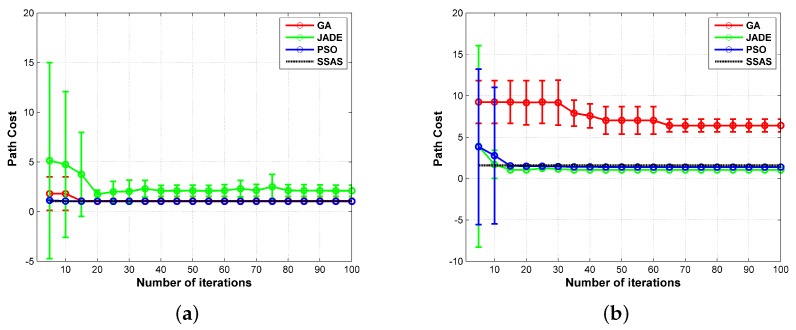
Convergence curves of the bio-inspired algorithms. (**a**) test case 1-s1; (**b**) test case 1-s4.

The best sub-paths designed by the three reference algorithms are displayed in Figure 21. The minimum, maximum, average, standard deviation (St Dev) of the path costs are listed in Table 7. These indices are calculated according to the results of all the successful experiments, in which a feasible route without constraints being violated is obtained at last. In addition, the success rate (SR) and the time each planner took in a single run are recorded in Table 7. The convergence curves of the three bio-inspired algorithms are given by Figure 22, which plots the mean and the stand deviation of the path cost. The curves in Figure 22 are calculated in the experiments with the best five results. It can be noted that all three of the bio-inspired algorithms converged to a solution after 100 iterations. As shown by Table 7, all three of the reference planners designed a feasible sub-routes successfully. The best costs of JADE, PSO and SSAS are very close to each other. Among them, the GA based method presents the worst performance in path cost. In addition, the routes planned by GA are not very stable as shown by the large values in its worst cost and standard deviation. However, the GA based planner took the least time, and its SR is better than the JADE based method. PSO presents the best performance but took the most time in a single run. The efficiency of SSAS can be seen clearly in this experiment. Taking the best outcomes of the three stochastic planners as the reference, it achieves the approximate performance but with much less runtime. When it comes to the second sub-path (see Figure 21b), the three reference planners show much worse performance. Although GA and PSO managed to provide feasible routes, their success rates are very low in this experiment. The success rate of GA is the highest, but its best sub-path costs more than three times of the one designed by SSAS. With the least value in the minimum cost, the PSO planner only succeed six times in 100 runs. That is to say, 17 runs are required to obtain a feasible route in average. With the time in each run being 11.2256 s, the time for a successful run is 187.1 s. Therefore, it is not suitable for the real-time implementation. In terms of the JADE planner, it failed to design a feasible route between the two vectors in 100 runs. By contrast, SSAS’s performance is more reliable. Although SSAS designed a longer path than the PSO planner, the time it took is only 5.6617 s, which is acceptable for real-time implementation.

## 6. Discussion and Conclusions

For an airborne reconnaissance system, a well-designed path is of great significance to enhance its reconnaissance efficiency. This paper presents an image-based path planner for the coverage of multiple ROIs with a single lightweight UAV SAR. Rather than only concentrating on the UAV, we account for the unique requirements of the SAR system on the motion of its platform in the path planning process. First, the problem under study is built as a constrained MOP. The constraints and objectives, either SAR-oriented or UAV-oriented, are analyzed in an image-based C-space. Second, morphological and contour analysis techniques are implemented to design proper path segments for data collection of the interested regions. Lastly, an effective and efficient route planning algorithm named SSAS is applied to connect the collection segments. The proposed SSAS could design sub-optimal routes with comparable costs at much less time compared with the stochastic router planners. Meanwhile, the construction of search map can be avoided by adopting the sampling strategy. Simulation experiments in real scenarios demonstrate that the proposed path planner could make the lightweight UAV SAR system operate more efficiently in conducting the surveillance mission. Furthermore, the time it takes is acceptable for real-time application.

To achieve a quick solution, the proposed path planner relaxes the constrained optimization problem in many aspects:

1. The flight altitude is assumed to be constant while the changing elevation of search area is not accounted for.
2. The ROIs are of comparable size to the radar swath width, and no more than one turn is required to achieve the full coverage of interesting regions.
3. The threats between ROIs are ignored when searching the visiting sequence and the approach angles. In some cases, the threats between the interesting regions may force the actual path to be curvy, which would prolong the path.
4. The threats are ignored in defining the feasible angles. In some cases, a threat close to an ROI could restrict the feasibility of some approach angles.
5. The coverage of some irregularly shaped ROIs can be achieved in a more economical way. For example, both legs of an L-shaped ROI can be traversed independently to save time and power.

These relaxations restrict the application of the proposed method in some cases, and the corresponding solutions will be studied in the future.

Another important issue in our future work is to extend our path planner to more complex scenarios, e.g., a 3D battlefield with more threats and interested targets. Furthermore, more operation modes of the SAR system such as the spotlight and ScanSAR modes could be considered. Lastly, a single UAV SAR is insufficient with a number of ROIs to be covered. Therefore, the cooperative route planning for multiple UAV SAR systems could be studied in the future.

## Figures and Tables

**Table 1:** SAR system parameters.

Description	Symbol	Value
Flight altitude	h	500 m
Incidence angle	$\theta_{L}$	45°
3 dB beam width (azimuth)	$\theta_{A}$	10°
3 dB beam width (elevation)	$\theta_{B}$	30°
Synthetic aperture length	$l_{s}$	123.41 m
Swath width	$l_{sw}$	577.35 m
Near-end distance	$l_{near}$	228.68 m

**Table 2:** Categories of ROIs in Figure 13c.

Type	Point ROI	Quasi-Point ROI	Distributed ROI
Label	R6	R2, R3, R5	R1, R4

**Table 3:** Description of test cases.

Case	Starting Point	Ending Point	Missile Centers	Radar Centers
1	(125, 234)	(1380, 880)	(336, 274) (785, 1294) (980, 1878) (1250, 1904) (968, 700) (418, 210)	(536, 478) (1070, 1302) (305, 1204)
2	(125, 234)	(1268, 2080)		
3	(1086, 129)	(116, 2080)		
4	(80, 1020)	(68, 1049)		

**Table 4:** Computational time of the proposed path planner in different cases.

	Case 1	Case 2	Case 3	Case 4	Case 5	Case 6	Case 7	Case 8
Step 2	16.475 s	16.406 s	16.181 s	16.633 s	42.112 s	42.339 s	61.806 s	61.925 s
Step 3	10.860 s	10.347 s	10.669 s	10.900 s	15.784 s	14.983 s	20.741 s	21.066 s
Step 4	45.490 s	26.795 s	22.796 s	**121.086 s**	89.839 s	**120.726 s**	**123.056 s**	95.012 s
Total	72.825 s	53.548 s	49.646 s	148.619 s	147.735 s	178.048 s	205.603 s	178.003 s

**Table 5:** Performance of the proposed and the zigzag path planners.

Case	Our Path			Vertical Zigzag Path			Horizontal Zigzag Path		
	RL	CL	DC	RL	CL	DC	RL	CL	DC
1	31,523.5 m	6206.2 m	19.69%	51,084.7 m	7879.1 m	15.42%	47,319.4 m	7803.4 m	16.49%
2	27,172.0 m	6058.6 m	22.30%	56,896.8 m	7879.1 m	13.85%	41,444.2 m	8008.9 m	19.32%
3	28,486.0 m	6546.6 m	22.98%	57,059.8 m	7879.1 m	13.81%	41,942.5 m	8149.5 m	19.43%
4	33,372.1 m	6392.7 m	19.16%	53,488.5 m	7879.1 m	14.73%	46,505.0 m	8008.9 m	17.22%

**Table 6:** Parameter settings of the stochastic path planning algorithms.

Planner	Parameters
GA [9]	$N_{p}=30,N_{s}=12,P_{c}=0.75,P_{m}=0.008$, $C_{ms}=0.1,C_{mb}=0.5,N_{W}=6$
JADE [43]	$N_{p}=10,N_{W}=6$
PSO [54]	$N_{p}=128,\omega =0.7298,c_{1}=c_{2}=1.4960,N_{W}=6$

**Table 7:** Performance comparison with the stochastic path planning algorithms.

	Algorithm	Min Cost	Max Cost	Mean Cost	St Dev	SR (%)	Runtime (s)
Test case1-s1	GA	1.0525	42,817.0624	978.7960	5430.2681	84	1.5981
	JADE	1.0456	13.2693	6.5973	2.7943	58	10.8631
	PSO	1.0302	6.5710	2.1465	2.0184	94	12.8228
	SSAS	1.0474			∖	∖	1.5076
Test case1-s4	GA	5.7333	60.0454	14.6081	13.4047	31	1.5665
	JADE	N/A	N/A	N/A	N/A	0	10.3604
	PSO	1.3592	1.4194	1.3844	0.0240	6	11.2256
	SSAS	1.5892			∖	∖	5.6617

## Abbreviations

The following abbreviations are used in this manuscript:

UAV	Unmanned Aerial Vehicle
SAR	Synthetic Aperture Radar
ROI	Regions of Interest
MOP	Multiobjective Optimization Problem
SSAS	Sampling-based Sparse A* Search
EA	Evolutional Algorithm
PSO	Particle Swarm Optimization
SAS	Sparse A* Search
DEM	Digital Elevation Map
PLR	Path Length Ratio
RKill	Risk of Kill
RRD	Risk of Radar Detection
MBR	Minimum Bounding Rectangle
TSP	Traveling Salesman Problem
WSM	Weighted Sum Model
RL	Route Length
CL	Collection Length
DC	Duty Cycle
GA	Genetic Algorithm
DE	Differential Evolution
SR	Success Rate
